# Right Pulmonary Artery Originating from Ascending Aorta (Hemitruncus Arteriosus) with VACTERL Association in a Neonate: A Case Report

**DOI:** 10.3390/children9020194

**Published:** 2022-02-03

**Authors:** Byeong-Su Shin, Taehong Kim, Hyoung Doo Lee, Hoon Ko, Joung-Hee Byun

**Affiliations:** Department of Pediatrics, Pusan National University Yangsan Hospital, Yangsan-si 50612, Korea; hermitmd1@gmail.com (B.-S.S.); hdlee@pusan.ac.kr (H.D.L.); peddrkh@gmail.com (H.K.); africa3217@naver.com (J.-H.B.)

**Keywords:** VACTERL association, anomalous origin of pulmonary artery, neonate

## Abstract

Vertebral, anal, cardiac, tracheo-esophageal fistula, renal and limb (VACTERL) association is defined as a condition including at least three of the above-mentioned anomalies in the same infant. Several cardiac defects that have been reported as a part of the VACTERL association are ventricular and atrial septal defects, hypoplastic left heart syndrome, transposition of the great arteries and tetralogy of Fallot. Anomalous origin of pulmonary artery (AOPA) from the ascending aorta is an unusual and critical cardiovascular anomaly, which frequently involves the right pulmonary artery (RPA). A male neonate was delivered by normal spontaneous vaginal delivery at 39 weeks and 3 days gestation, weighting 2660 gm. He was diagnosed with VACTERL association with five abnormalities: vertebral abnormalities, anal atresia, cardiovascular anomaly (right pulmonary artery originating from ascending aorta), tracheo-esophageal fistula and renal anomalies. AOPA origination from ascending aorta as part of the VACTERL association in a neonate is a rare congenital cardiovascular malformation. Here we present a rare case of RPA originating from the ascending aorta seen with VACTERL association in a neonate.

## 1. Introduction

Vertebral, anal, cardiac, trachea-esophageal fistula, renal and limb (VACTERL) association is a combination of congenital malformations involving multiple body systems. Diagnosis of VACTERL association requires any three of the following findings: vertebral abnormalities, anal atresia, heart anomalies, tracheo-esophageal fistula, and real and limb defects [1]. The prevalence at birth varies from 1 in 10,000 to 1 in 40,000 newborns [2]. The most common heart defects in VACTERL association are ventricular septal defects (VSD), atrial septal defects (ASD), hypoplastic left heart syndrome (HLHS), transposition of the great arteries (TGA) and tetralogy of Fallot (TOF) [3].

Anomalous origin of the pulmonary artery (AOPA) from the ascending aorta (Ao) (also known as hemitruncus arteriosus) is a relatively uncommon and a very serious cardiovascular anomaly, which more frequently involves the right pulmonary artery (RPA) than the left pulmonary artery [4]. The overall incidence of AOPA from the Ao is 0.1% of all congenital cardiac diseases and has very high mortality rate in the first year [5,6]. To prevent the development of irreversible pulmonary vascular disease and for improved survival rate, early diagnosis and surgical repair of AOPA in a neonate is important.

To our knowledge, cases of AOPA from the ascending aorta have rarely been reported with VACTERL association. Here we present a case of RPA originating from the Ao, which is an uncommon congenital cardiovascular anomaly, as part of the VACTERL association in a neonate.

## 2. Case Report

A male neonate was delivered by normal spontaneous vaginal delivery at 39 weeks and 3 days gestation, weighting 2660 gm (10~25th percentile). His mother’s obstetric history was gravid 1, para 0. There was no history of familial diseases. Prenatal ultrasonography of the patient revealed single umbilical artery, left renal pelvic ectasia and suspicion of fetal anal atresia.

At birth, he had mild respiratory difficulty. His APGAR scores were 6 and 5 points at 1 and 5 min respectively. His height was 45.5 cm (10th percentile), and head circumference was 35 cm (75~90th percentile). On examination, his blood pressure was 68/44 mmHg, heart rate was 124 bpm, and respiratory rate was 30 breaths/min. He had a single umbilical artery, and the anus was imperforate. A nasogastric tube was inserted, which was not advanced. Oxygen was administered at a flow rate of 2 L/minute using a nasal cannula due to tachypnea and low SpO_2_ (85%) in room air. Despite of nasal oxygen inhalation, he had severe sialorrhea, dyspnea, low SpO_2_ (60%) and was subsequently intubated and placed on mechanical ventilation.

**Figure 1:**
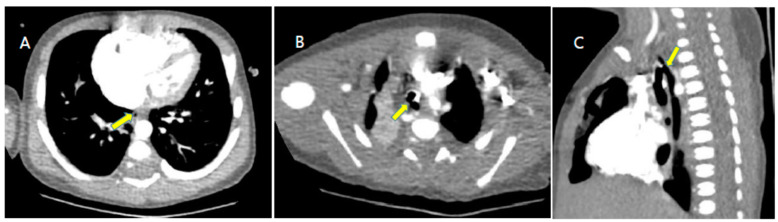
Chest computed tomography (CT). (**A**): Chest CT image exhibiting esophageal proximal blind end (arrow). (**B**): cross-section view, (**C**): lateral view Fistula (arrow) connecting proximal esophagus with trachea, tracheo-esophageal fistula at the level of thoracic vertebrae 3-4.

**Figure 2:**
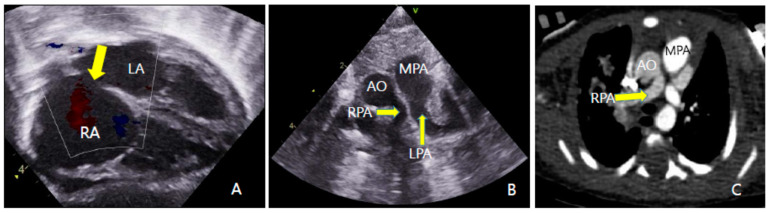
Echocardiographic image (**A**,**B**) and CT angiogram (**C**). (**A**): Subcostal image showed atrial septal defect (arrow) (LA: left artirum; RA: right atrium). (**B**): Parasternal short-axis image demonstrated that RPA originated from the aorta and LPA originated from the MPA. (**C**): CT angiography image demonstrated that RPA arose from the aorta. (MPA: main pulmonary artery; AO: ascending aorta; RPA: right pulmonary artery; LPA: left pulmonary artery).

**Figure 3:**
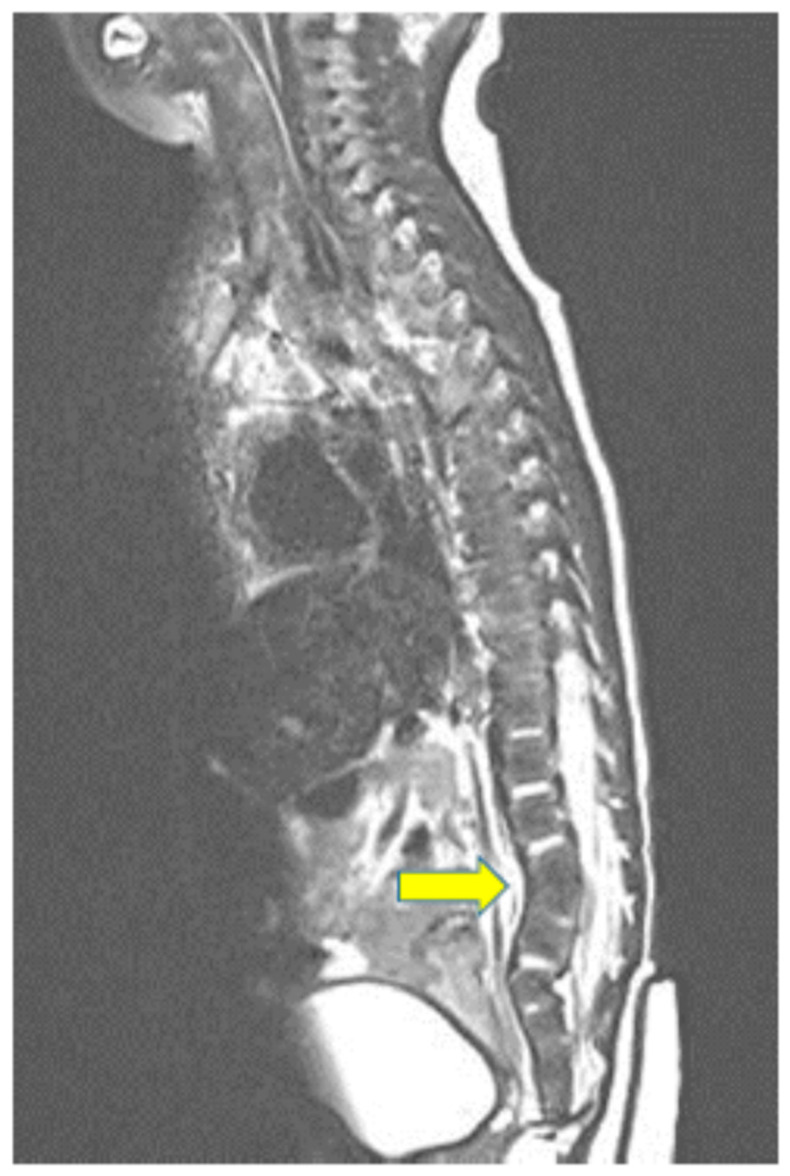
Sagittal T2-weighted spine magnetic resonance image demonstrates block vertebrae L4-5 (arrow) and sacral dysgenesis.

**Figure 4:**
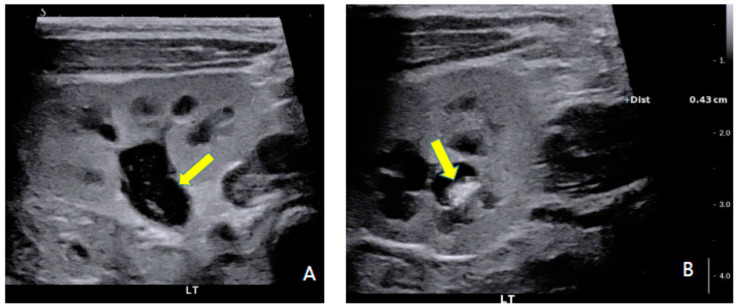
Renal ultrasonography showed pelvocalyceal system dilatation (arrow) of left kidney (**A**) and stone (arrow) in the lower pole of the left kidney (**B**).

Chest computed tomography (CT) showed a fistula connecting the proximal esophagus and the trachea at the level of thoracic vertebrae 3-4, which was diagnosed as tracheo-esophageal fistula (Figure 1). He had a 6 mm sized ASD, 5 mm sized patent ductus arteriosus (PDA) and the RPA originated from the Ao on echocardiography and chest CT (Figure 2). A sagittal T2-weighted spine magnetic resonance image revealed block vertebrae L4-5 and sacral dysgenesis (Figure 3). Renal ultrasonography showed urinary tract dilatation of the left kidney and 0.4 cm sized stone in the lower pole of the left kidney (Figure 4). Chromosomal microarray analysis was obtained and showed normal male karyotype.

He was diagnosed with VACTERL association with five anomalies, specifically vertebral malformations, anal atresia, cardiovascular anomalies (anomalous origin of RPA originating from Ao), tracheo-esophageal fistula, and renal anomalies.

On the second day after birth, he underwent sigmoid-loop colostomy due to imperforated anus (high type) and on the sixth day of life, a primary anastomosis of esophagus and excision of trachea-esophageal fistula was performed. A cardiac surgeon performed total correction of the patient’s heart anomalies (detachment of RPA from Ao, anastomosis of the RPA to main pulmonary artery and direct closure of ASD) at 14 days of age. After surgical repairs, he was treated with antibiotics due to urinary tract infection and underwent esophageal balloon dilatation for esophageal stricture of the surgical site. He was finally discharged with oral feeding at the age of 2 months.

## 3. Discussion

In the early 1970s, vertebral defects, anal atresia, T-E fistula with esophageal atresia, radial and renal dysplasia was first reported by Quan and Smith. As initially described, the association regarded the statistically nonrandom concurrence of particular congenital abnormalities [7]. Within the next 2 years, several case reports suggested that single umbilical artery and cardiac anomalies should be included as part of the VATER/VACTERL association [3].

The estimated frequency varies from 1 in 10,000 to 1 in 40,000 newborns. The true frequency may be difficult to determine because different diagnostic criteria have been used in different studies [2,8]. The exact etiology of VACTERL association is not clear. Quan and Smith [7] suggested that the common defect in early differentiating mesoderm during embryogenesis causes VACTERL association. Recently, mitotic mosaic clone elimination, that is, the clone elimination-related development disruption at specific locations, followed by mitotic mosaic aneuploidy, has been suggested as the basic etiology of the VACTERL association [9]. Only a small percentage of patients with VACTERL association have been associated with genetic causes, such as those with mitochondrial dysfunction, or copy number variations (gene alterations including deletions or duplications) [10]. In our case, reports of chromosomal microarray analysis were normal.

The general prevalence of component findings of VACTERL association is 60~80% in vertebral defects, 55~90% in anal atresia, 40~80% in cardiac anomalies, 50~80% in trachea-esophageal fistula with/without esophageal atresia, 50~80% in renal anomalies, and 40~50% in limb anomalies [2,11]. Our patient had five anomalies, vertebral anomalies, anal atresia, cardiovascular defects, tracheo-esophageal fistula and renal abnormalities.

A number of different cardiovascular anomalies may occur in the VACTERL association, the most common being VSD. Additional congenital cardiac anomalies that have occurred in the VACTERL association include ASD, HLHS, TOF, and TGA [3]. Our patient had ASD and RPA originated from Ao (also known as hemitruncus arteriosus).

AOPA from the Ao is an uncommon and critical cardiovascular defect, which frequently involves the RPA. Three sites of origin for AOPA have been reported: a proximal one, in which the AOPA originates from the posterolateral wall of Ao above the aortic sinus, a distal one in which AOPA arises via a PDA, and a third form in which originate distally from the Ao near the origin of the innominate artery [12]. In our case, RPA originated from the left posterior wall of the Ao above the sinotubular junction.

Fraentzel published the first report in 1868. The frequency of AOPA is 0.1% of all congenital cardiac disease [13]. In AOPA, all of the oxygen-poor blood from the right ventricle supplies to one lung and the other lung is supplied with oxygenated blood from the left ventricle, which circulates back to the left atrium. This oxygen-poor volume flow to the one lung causes progressive worsening of pulmonary vascular disease and right ventricular hypertrophy. In contrast, the other lung receives oxygenated flow through the anomalous pulmonary artery, and it too develops pulmonary vascular disease. With the development of the disease, pulmonary arterial hypertension and congestive heart failure often occur within one year after birth. Irrespective of the circulation, early diagnosis and surgical correction is important to prevent the development of irreversible pulmonary vascular disease. Infants who do not have surgical repair have a mortality rate up to 70% one year after birth, and 30% of infants die within 3 months [14]. In our case, the patient underwent surgical correction at 14 days.

VACTERL association is a relatively uncommon finding in clinical practice. AOPA is rare congenital heart anomaly, which has not been reported as part of the VACTERL association. This is the first reported incidence of the RPA originating from the Ao as a cardiac manifestation of the VACTERL association in a neonate.

## Data Availability

The data supporting the findings of this study are available from the corresponding author on reasonable request.
